# Wrist-worn device combining PPG and ECG can be reliably used for atrial fibrillation detection in an outpatient setting

**DOI:** 10.3389/fcvm.2023.1100127

**Published:** 2023-02-09

**Authors:** Harri Juhani Saarinen, Atte Joutsen, Kirsi Korpi, Tuomas Halkola, Marko Nurmi, Jussi Hernesniemi, Antti Vehkaoja

**Affiliations:** ^1^Heart Hospital, Tampere University Hospital, Tampere, Finland; ^2^Faculty of Medicine and Health Technology, Tampere University, Tampere, Finland; ^3^Department of Medical Physics, Tampere University Hospital, Tampere, Finland; ^4^Finnish Cardiovascular Research Center, Tampere University, Tampere, Finland; ^5^PulseOn Oy, Espoo, Finland

**Keywords:** atrial fibrillation, wearable sensors, photoplethysmography, electrocardio graphy, ambulatory

## Abstract

**Aims:**

The aim was to validate the performance of a monitoring system consisting of a wrist-worn device and a data management cloud service intended to be used by medical professionals in detecting atrial fibrillation (AF).

**Methods:**

Thirty adult patients diagnosed with AF alone or AF with concomitant flutter were recruited. Continuous photoplethysmogram (PPG) and intermittent 30 s Lead I electrocardiogram (ECG) recordings were collected over 48 h. The ECG was measured four times a day at prescheduled times, when notified due to irregular rhythm detected by PPG, and when self-initiated based on symptoms. Three-channel Holter ECG was used as the reference.

**Results:**

The subjects recorded a total of 1,415 h of continuous PPG data and 3.8 h of intermittent ECG data over the study period. The PPG data were analyzed by the system’s algorithm in 5-min segments. The segments containing adequate amounts, at least ~30 s, of adequate quality PPG data for rhythm assessment algorithm, were included. After rejecting 46% of the 5-min segments, the remaining data were compared with annotated Holter ECG yielding AF detection sensitivity and specificity of 95.6 and 99.2%, respectively. The ECG analysis algorithm labeled 10% of the 30-s ECG records as inadequate quality and these were excluded from the analysis. The ECG AF detection sensitivity and specificity were 97.7 and 89.8%, respectively. The usability of the system was found to be good by both the study subjects and the participating cardiologists.

**Conclusion:**

The system comprising of a wrist device and a data management service was validated to be suitable for use in patient monitoring and in the detection of AF in an ambulatory setting.

**Clinical Trial Registration**: ClinicalTrials.gov/, NCT05008601.

## 1. Introduction

Atrial fibrillation (AF) is the most common arrhythmia diagnosed in clinical practice with increasing numbers forecast due to the worldwide aging of large generations (1) AF results from chaotic activation of multiple origins in the atrial muscle of the heart. AF predisposes patients to embolic stroke and anticoagulation medication should be considered if the CHA_2_DS_2_VASc score of the patient is one or higher. One in three to four patients with ischemic stroke, and over 80% of those with ischemic stroke of cardioembolic type, also had atrial fibrillation (2). There is also evidence of an association between AF and cognitive dysfunction ranging from mild impairment to overt dementia (3, 4). This makes recognizing and diagnosing AF critical. On the other hand, the brief paroxysms of AF can be very difficult to detect, and in some patients, AF may be asymptomatic. The European Society of Cardiology guidelines for the diagnosis and management of AF recommend that a minimum of 30 s single-lead electrocardiogram (ECG) with irregular rhythm without discernible repeating P waves is required for the diagnosis of AF (5, 6).

Inexpensive, convenient, and reliable means to diagnose AF could improve the prevention AF related stroke and death and also the development of cognitive dysfunctions. The paroxysmal nature of AF episodes may limit the use of conventional 12-lead ECG recorded on demand by health care practitioners. Implantable loop recorders are better suited for other purposes than diagnosing AF as they are invasive and expensive. Modern wearable devices such as smart watches and smart phones can be used to screen the heart rhythm for anomalies almost continuously (smart watches) or intermittently (smart phones) *via* photoplethysmography (PPG), but recorded ECG is still required for a diagnosis. Some of the new smart watches feature both optical cardiac rhythm monitoring and a capability to record a single-lead ECG tracing. However, certified medical devices featuring both modalities and intended for clinical use have so far been lacking.

The objective of this study was to validate the performance of the PulseOn Arrhythmia Monitor System (PulseOn Oy, Espoo, Finland) consisting of a wrist-worn device and a data visualization cloud service, the PulseOn Data Management Service, intended to be used by medical professionals in detecting AF in an outpatient setting for 48 h.

## 2. Materials and methods

The intended purpose of the PulseOn Arrhythmia Monitor System is to assist in the diagnosis, screening, and monitoring of cardiac arrhythmias, especially atrial fibrillation. The system consists of a wrist-worn device and a secure cloud-based data management service. The wrist device optically monitors the user’s pulse rate to detect any heartbeat irregularity and is used to take intermittent single-lead (Lead I) ECG measurements between the arms. The wrist device stores the measured data, which is later transferred to the data management service where it can be analyzed by medical professionals. The device is intended to be used inside and outside the hospital environment. The usage period of the system may vary from days to several weeks. A descriptive, observatory clinical investigation was conducted to validate the system’s performance (clinical trial NCT05008601).

### 2.1. Study population

According to the performance results of earlier clinical feasibility studies, an estimated half of the obtained data was sinus rhythm and the other half AF data. Using 0.1 as the probability of type I error and 80% power level, the required amount of data was estimated as 500 h based on a non-inferiority approach (7). However, due to several uncertainties in estimated values, the target subject number was set at 30 instead of the minimum 11 for 48-h recordings.

**Figure 1:**
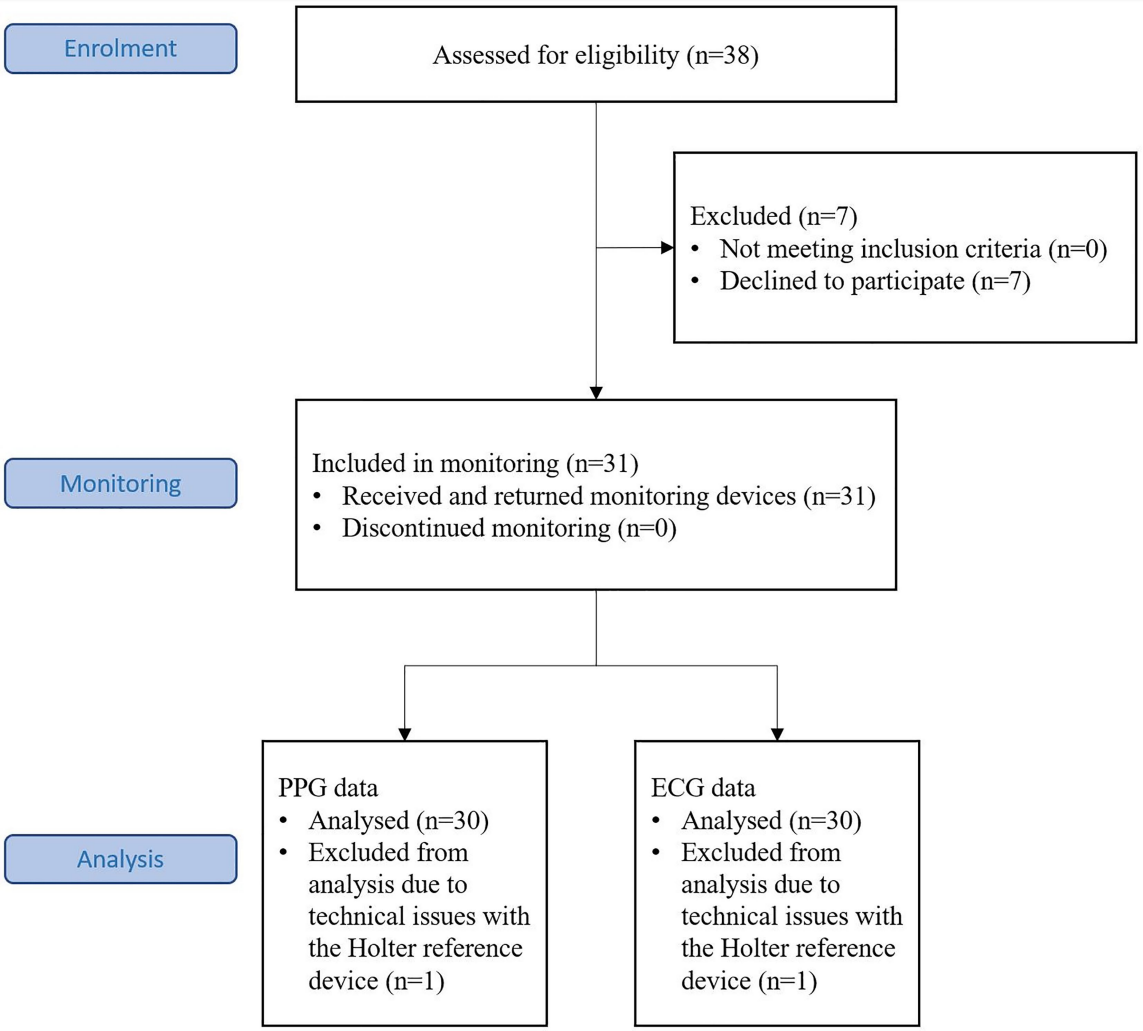
The Consort flow diagram for the trial.

The targeted subject number was based on the primary study objective of showing the sensitivity of the PPG based arrhythmia detection algorithm in detecting atrial fibrillation when the analysis is made in 5-min windows. Subjects were recruited who met the inclusion criteria: age ≥ 18 years and prior diagnosis of AF alone or AF with concomitant atrial flutter were recruited. Patients with pacemakers were excluded. Thirty-eight subjects were assessed for eligibility, seven were excluded and 31 included in the monitoring (Figure 1).

### 2.2. Wrist device and data management service

**Figure 2:**
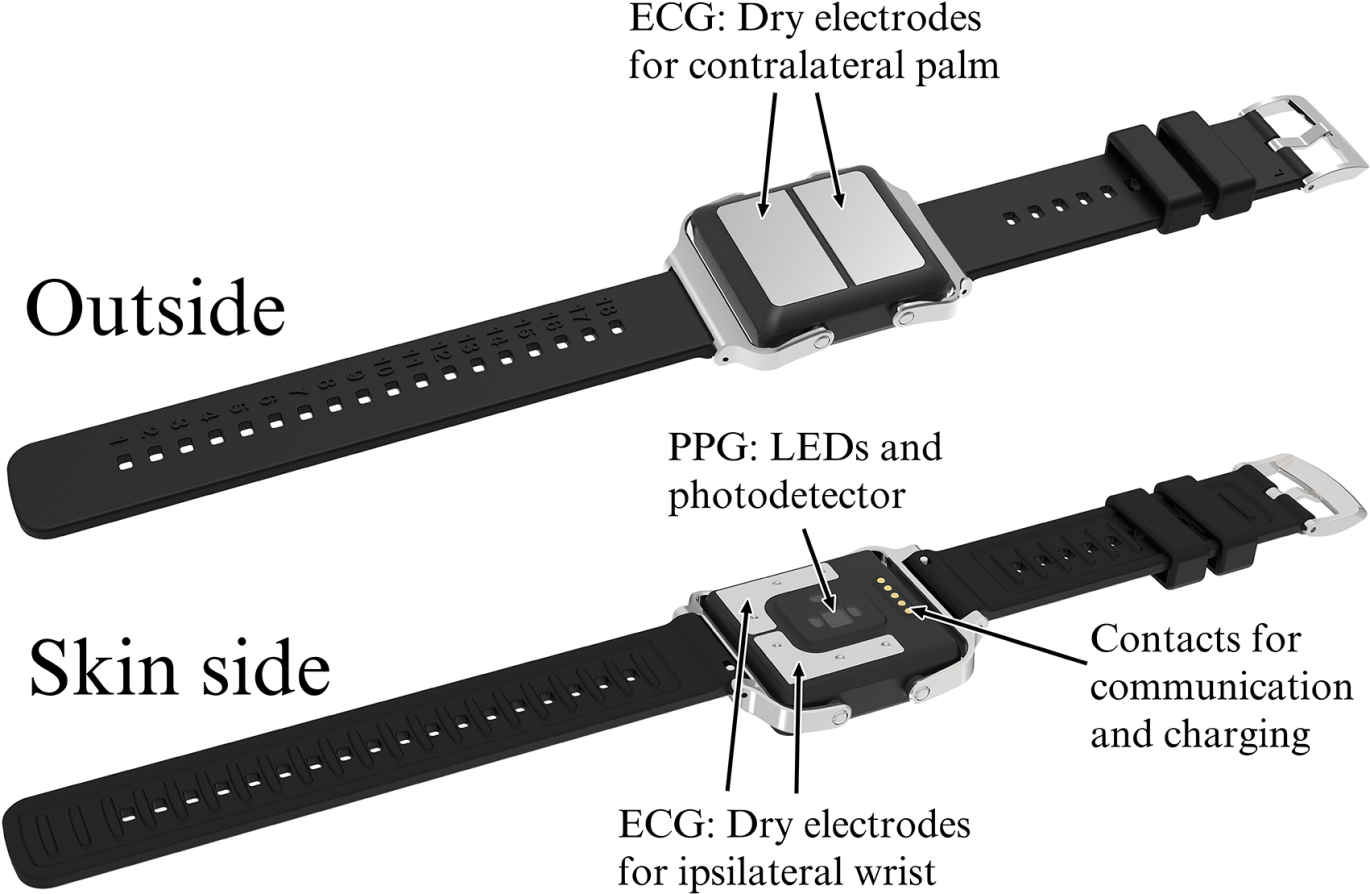
The PulseOn Arrhythmia Monitor wrist device outside (above) and skin side (below) with details.

The wrist device used in the PulseOn Arrhythmia Monitor System includes both reflective mode wrist PPG with yellow-colored LEDs as well as stainless steel dry electrodes to enable Lead I ECG measurement when the recording loop is closed by placing the contralateral palm on the wrist device (Figure 2). The device continuously measures PPG from the patient’s wrist to analyze the beat-to-beat heart rate for possible cardiac rhythm irregularities. When irregular rhythm is detected, the device notifies the patient to take a 30-s ECG recording for further analysis. The exact duration of the recording is 35 s of which 30 s are shown to the healthcare professional. The notifications can also be scheduled to take place 1–4 times a day. In addition, the patient can self-initiate recordings whenever there is a need, e.g., if they experience arrhythmia symptoms. The PPG-based inter-beat-intervals (IBI), the heart rhythm status based on the IBIs and recorded ECGs are stored in the internal memory of the wrist device. In normal operation, the wrist device can store up to 6 months of data to its internal memory.

**Figure 3:**
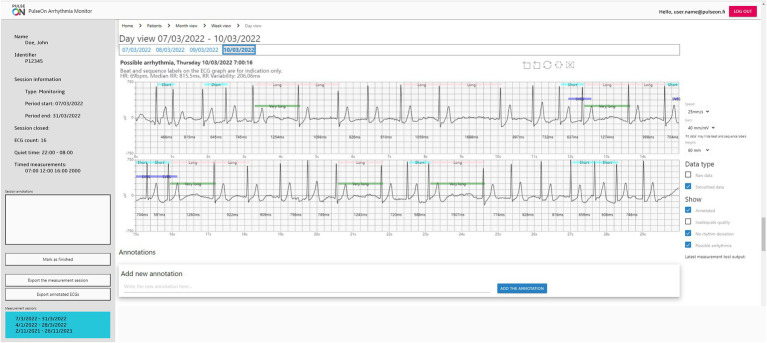
ECG view of PulseOn Data Management Service.

The data can be transferred *via* a gateway to a server or, as in this study, be downloaded at the clinic when the wrist device is returned. The data analysis and patient rhythm assessment are done by the PulseOn Data Management Service through a web browser user interface. The Data Management Service includes ECG analysis algorithms (Cardiolund AB, Lund, Sweden) that process the measured ECG signals and flag signals showing signs of arrhythmia (Figure 3). The service features three views: a monthly overview, a more detailed weekly view, as well as an ECG signal view. The ECG view shows the measured ECG signals including beat specific markings overlaid on the signal, RR-intervals in milliseconds and the labeling of each recording made by the algorithms. The markings include *Short*, *Long and Very long RR-intervals, Supraventricular extrasystoles (SVES), Ventricular extrasystole (VES), Tachycardia, Fast, Slow*, Bigeminy, and Trigeminy. The labels for the whole 30-s record comprise *Possible arrhythmia, Inadequate quality, No rhythm deviation, Pause/AVblock II, Fast regular, Fast regular and wide QRS, Fast/Slow episode, Bigeminy, Trigeminy, Wide QRS, > 5 SVES, and > 5 VES*.

The device and the silicone wrist strap are easy and quick to clean between patients using common cleaning agents. The device is classified as waterproof up to 1 m and the battery lasts for more than 7 days without recharging. In longer studies, the patient is given an easy-to-operate charging dock.

This current validation was performed for the CE approval of the PulseOn Arrhythmia Monitor System as a class IIa medical device for its intended purpose according to the regulation (EU) 2017/745 of the European Parliament and of the Council on Medical Devices.

### 2.3. Data collection

The subjects were asked to simultaneously wear two different devices for cardiac rhythm monitoring: the PulseOn wrist device and a three-channel Holter device (Faros 360, Bittium Biosignals Oy, Oulu, Finland) with disposable Ag/AgCl gel electrodes (Ambu Blue Sensor L-OO-S, Ambu A/S, Ballerup, Denmark). The Holter device was used to obtain reference information on the heartbeat intervals and rhythm status of the subjects. The subjects wore the devices continuously during the 48-h study period. Six individual devices of both types were circulated among the subjects. Data collection was started during an outpatient visit. The data were collected during the subjects’ normal daily activities.

In addition to the continuous PPG recording of heartbeat intervals, the subjects were instructed to collect 30-s ECG recordings in three cases: first, if the device gave a timed reminder to take a recording (four times a day at 8:00, 12:00, 16:00, and 20:00); second, if the device gave an arrhythmia notification based on the PPG monitoring; and third, if the subject experienced arrhythmia symptoms. Thus, at least four intermittent ECG recordings were taken daily.

### 2.4. Signal analysis

Two experienced cardiologists investigated the collected ECG data. The reference Holter-recordings were annotated by a cardiologist (HJS) blind to the wrist device data using Darwin2 Holter analysis software (Schiller Americas, Doral, FL, United States). In addition to the standard hour by hour statistical Holter report, the precise time points for the beginning and ending of the arrhythmia episodes were marked and used in the estimation of the sensitivity and specificity of both the automated PPG and the ECG analysis algorithms. Another cardiologist (KK) assessed the cardiac rhythm from the wrist device ECG recordings blind to the reference data. HJS also assessed the rhythm using the wrist device ECG recordings. This assessment was done after a significant amount of time (approximately 6 months) had elapsed since annotating the Holter-recordings to retain objectivity regarding the assessment. The cardiologists’ wrist device ECG appraisals were used for data quality assessment and to determine how many subjects showing AF in the Holter-recordings could be correctly classified visually using only the wrist device data.

The evaluation of the PPG-based arrhythmia detection was made using 5-min data segments. The 5-min analysis window length has earlier been used by Zhang et al. (8) and Chang et al. (9). If more than 30 consecutive heartbeats were classified as arrhythmia or regular rhythm, the whole segment was appropriately labeled as arrhythmia or regular rhythm, arrhythmia having priority if both rhythm types were found in the segment. Those 5-min segments during which the arrhythmia analysis algorithm had not been able to make a rhythm assessment, e.g., due to too much movement, were labeled as undetermined and excluded from the sensitivity/specificity analysis. The same 30 consecutive heartbeat threshold is used by the wrist device to give irregular rhythm notification.

### 2.5. Usability

After the 48-h recording, the subject returned the wrist device and completed a usability questionnaire. Fourteen items on the questionnaire included the subject’s impressions of the clarity of the device’s notifications (four items), comfort when wearing the device (six items), possible skin irritation (one item), ease of recording a 30 s ECG (two items), and an overall grade for the device. The scale was 1–5 in 10 questions and binary (Yes/No) in four questions. Open-ended comments were invited to supplement the structured questions.

The cardiologists KK and HJS were interviewed about how they felt about using the PulseOn Data Management Service for reviewing the wrist device ECG data.

### 2.6. Statistical analysis

The performance of the PulseOn Arrhythmia Monitor System was assessed by comparing the wrist device PPG segments and ECG records labeled by the wrist device and Data Management Service algorithms with the cardiologists’ Holter ECG manual annotations. The wrist device data were scored as seen in Table 1. Sensitivity and specificity were calculated from these results.

**Table 1:** The data labels used in scoring the wrist device’s data.

Label in ECG	Label in PPG	Label in Holter	Result
Possible arrhythmia	Arrhythmia	Atrial fibrillation	True positive
Possible arrhythmia	Arrhythmia	Sinus rhythm	False positive
No rhythm deviation	Regular rhythm	Sinus rhythm	True negative
No rhythm deviation	Regular rhythm	Atrial fibrillation	False negative

The accuracy of the IBIs estimated from the PPG signal was evaluated by first aligning the IBIs with the reference RR-intervals obtained from the ECG signal and then calculating the average of the absolute of the difference between the corresponding intervals. This metric is often called mean absolute error. Only the IBIs marked “reliable” by the PPG analysis algorithm were considered.

### 2.7. Consent and ethical considerations

The study followed the ethical principles of the Declaration of Helsinki, and each study subject gave written informed consent. The study protocol was approved by the Research Ethics Board of Tampere University Hospital (decision number R20087) and the national competent authority Fimea. The study was registered in the open clinical trial database ClinicalTrials.gov (NCT05008601).

## 3. Results

Thirty-one volunteer subjects aged from 32 to 83 years were recruited from the patient base of Tampere Heart Hospital, Finland. One of the subjects was excluded after the data collection because of a technical problem with the reference Holter data (Figure 1). Two recordings were terminated prematurely at 31 and 32 h of data collection due to wrist device software failure. The recordings until the time of termination were reviewed, found to be intact, and included in the material. Of the final 30 subjects eight were female and 22 were male. The median age was 65 (IQR: 57–71) years. The diagnoses were: Paroxysmal AF: 21 subjects, Persistent AF: three subjects, Chronic AF: one subject, Paroxysmal AF and Typical AFL: three subjects, Persistent AF and Typical AFL: one subject, and Unspecified AF and AFL: one subject. The recordings were performed between March and June 2021. None of the subjects was hospitalized during the study period.

A total of 1,416 h of wrist device data were collected from the 30 subjects included, corresponding on average to 47 h 12 min per subject. Reference Holter ECG data were collected simultaneously, and totaled 1,438 h. Based on the cardiologists’ annotations, 150 h (10%) of the Holter data was of low but analyzable signal quality due to artifacts. Of the Holter data, 371.8 h (25.9%) showed AF and 50.7 h (3.5%) AFL. No adverse events occurred during the study.

### 3.1. PPG performance

The PPG dataset included 16,980 5-min data segments. Of these segments, 7,828 (46%) were labeled as *undetermined* by the algorithm due to inadequate data quality. Of all the day time (8:00–22:00), segments 69% were labeled as *undetermined*. In the night-time (22:00–8:00), only 21% were labeled as *undetermined*. The above segments were discarded from the sensitivity/specificity analysis. The remaining 9,152 segments (54%) were of adequate quality for rhythm analysis. Of these, 2,419 were labeled as arrhythmia and 6,733 as regular rhythm. After matching the annotated Holter ECG with the PPG, a sensitivity/specificity analysis was conducted for each subject and the whole data set (Table 2).

**Table 2:** Confusion matrix of PPG-based atrial fibrillation detection.

	Positive prediction	Negative prediction	Total
Actual atrial fibrillation	True positives 2,364	False negatives 110	2,474
Actual non-AF rhythm	False positives 55	True negatives 6,623	6,678
Total	2,419	6,733	9,152

The sensitivity and specificity of the 5-min based PPG atrial fibrillation detection were, respectively, 95.6 and 99.2%. The mean absolute error of the PPG inter-beat-interval estimation averaged over the whole dataset was 26.6 ms.

One study subject had a diagnosis of unspecified AF with concomitant atrial flutter whose rhythm was very stable most of the time but occasionally became irregular. This subject was considered to be non-AF in the analysis. Stable flutter rhythm is not detectable with PPG technology that utilizes heartbeat interval regularity analysis. The irregular rhythm periods however produced PPG-based arrhythmia notifications and caused 23 out of the total 55 *false positive* labels in Table 2.

### 3.2. ECG performance

The subjects recorded a total of 457 30-s ECG measurements using the wrist device. The number of ECG measurements per subject over the 48-h study period varied between 7 and 28. The prescheduled measurements were 47%, the PPG triggered were 23%, and the self-initiated were 30% of all the ECG segments.

Of the 457 30-s ECG segments, the algorithm labeled 44 (10%) as inadequate quality. These segments were excluded from the subsequent analysis. In addition, the cardiologists KK and HJS, respectively, labeled 24 (5%) and 5 (1%) of the remaining ECG segments as inadequate quality. These segments were included in the analysis of automatic ECG algorithm performance as the system as such was under evaluation. The algorithm identified 413 (90%) of the segments as analyzable quality. Based on the visual assessment of only the wrist device ECG data, the cardiologists were able to correctly classify all the subjects having episodes of AF during the measurement period. None of the subjects who were in sinus rhythm throughout the whole measurement period were incorrectly assessed as having arrhythmia episodes.

Fifteen subjects did not have any episodes of AF or AFL during the recording. Five of these had atrial tachycardia episodes of less than 30 s, thus not meeting the criteria for AF. Seven subjects had episodes of paroxysmal AF during the recording, seven subjects were in continuous AF and one in continuous AFL throughout the whole recording. However, both cardiologists found the aforementioned subject who had stable atrial flutter rhythm with periods of irregularities challenging to interpret. Flutter waves are often poorly visible in Lead I ECG making it difficult to assess atrial flutter (6). Neither of the cardiologists was confident about their analyses, however, assessing the subject as being either in AF or in mixed AF and flutter rhythm. On an ECG segment level, the algorithm labeled 197 segments as *Possible arrhythmia* indicating primarily AF and 215 either as *No rhythm deviation* indicating sinus rhythm or with any other of the labels listed below. Sensitivity and specificity were calculated based on the confusion matrix of Table 3 showing 97.7% sensitivity and 89.8% specificity.

**Table 3:** Confusion matrix of ECG-based AF detection.

	Positive prediction	Negative prediction	Total
Actual atrial fibrillation	True positives 173	False negatives 4	177
Actual non-AF rhythm	False positives 24	True negatives 211	235
Total	197	215	412

The Data Management System labels the 30-s ECG records with one of the following labels: *Possible arrhythmia, Inadequate quality, No rhythm deviation, Pause/AVblock II, Fast regular, Fast regular and wide QRS, Fast/Slow episode, Bigeminy, Trigeminy, Wide QRS, > 5 SVES, and > 5 VES*. In the above analysis only the label *Possible arrhythmia* was considered to indicate AF, which yields the results in Table 3. If any label, excluding *Inadequate quality* and *No rhythm deviation*, is considered to indicate AF, the sensitivity becomes 100.0% and specificity 84.26% (Table 4). This approach can be considered justified because in clinical use, the cardiac rhythm is always visually confirmed from the ECGs and the labels of the automatic analysis can be used attract the attention of the clinician performing the assessment.

**Table 4:** Confusion matrix of ECG-based cardiac arrhythmia detection.

	Positive prediction	Negative prediction	Total
Actual atrial fibrillation	True positives 177	False negatives 0	177
Actual non-AF rhythm	False positives 37	True negatives 198	235
Total	214	198	412

Further, if atrial flutter is considered together with atrial fibrillation the results become 99.5% sensitivity and 87.6% specificity.

### 3.3. Usability

Test subject feedback based on the questionnaire was received from 25 out of the 30 subjects. The average of the overall grade was 4.6 out of a maximum of five. The averages of the clarity of notifications was 4.2, wear comfort was 4.1, and ease of ECG recording was 4.6. The average of skin irritation was 1.4 (1 = no irritation, 5 = severe irritation). Thirteen percent of the subjects found notifications occasionally disturbing, 25% percent had to adjust the wrist strap tightness, 91% felt they knew what the correct tightness should be, and 4% felt uncomfortable and had to switch the device to another wrist. The comments in the subjects’ own words included generally positive remarks as well as comparisons, according to which the wrist device was preferred to the Holter.

Both cardiologists gave similar types of positive feedback on the usability of the PulseOn Data Management Service: the user interface of the Data Management Service supports the cardiologist’s work well, it is logically organized, has well-functioning review tools and the web browser interface responds promptly to the user’s commands. However, it should be noted that KK has had minor involvement in the design work of the Data Management Service and is a part-time employee of PulseOn Oy.

## 4. Discussion

The main finding of this study was that the wrist device investigated and the PulseOn Data Management Service evaluated can be reliably used to detect and diagnose AF in an ambulatory setting in daily life. The usability of the wrist device, comfort wearing it, and ease of ECG recording were rated good by the study subjects. Little to no skin irritation was experienced. The cardiologists found the Data Management Service to be well functioning for its purpose.

### 4.1. Photoplethysmogram

Our results in the PPG-based atrial fibrillation detection (sensitivity 95.6% and specificity 99.2%) are in line with those of earlier studies. Similar methodology has been previously used by four groups in five studies. Zhang et al. used the Samsung Galaxy Active 2 watch over 4 weeks on 53 patients and compared AF detection to continuous patch ECG. The sensitivity and specificity of the device were 90.8 and 93.0%, respectively, (8). Chang et al. recruited 200 participants who underwent 24 h of simultaneous Holter ECG monitoring and continuous PPG recording using a Garmin Forerunner 945 smartwatch. AF detection sensitivity and specificity were 97.1 and 86.8%, respectively, (9). The Philips Cardio and Motion Monitoring Module was used in two 24-h studies with 20 and 27 patients with Holter reference. The respective AF detection sensitivities and specificities of the two studies were 98.4 and 98.0% (10) and 100 and 96% (11). Wasserlauf et al., studied 24 patients who had an insertable loop recorder and wore an Apple Watch with Kardiaband during daytime for an average of 110 days. In their study, AF episodes of ≥1 h were detected with a sensitivity 97.5% per episode. Considering the total duration of all the AF episodes detected (loop recorder 1127.1 h, watch 1101.1 h) sensitivity and specificity were 97.7 and 98.9%, respectively, (12).

In our study, the PPG algorithm labeled 46% of the 5-min segments as *undetermined* because of inadequate data quality. The inadequate segments were recorded mostly in the day time. In the night-time, the artifacts were reduced as the subjects were resting. The amount of inadequate data is comparable with the previous studies: 42% calculated from Chang et al. (9), 24–57.6% as reported by Eerikäinen et al. (10) and 56% as reported by Bonomi et al. (11).

In addition to the above long-term studies with ambulatory outpatients, there have been several short-term studies with hospital inpatients. In these studies, the patients have been sitting or lying down, and are therefore not easily comparable with the free-living conditions of the outpatients. Nevertheless, the following results of the 21 studies reviewed reflect the current state of the art in using PPG for AF detection. Median number of patients was 60, median recording duration 10 min, median sensitivity 97.03% (range 84.10–100%) and specificity 96.00% (56.64–99.90%). (13–33)

The largest published studies on wrist-worn PPG devices and AF detection in normal daily living are those by Perez et al. using the Apple Watch on 419,297 (34) and Guo et al. using Huawei’s technology on 187,912 participants (35). In the Apple study, the detected AFs were subsequently confirmed by using ECG patches. In the Huawei study, the confirmation was by using clinical evaluation, electrocardiogram, or 24-h Holter monitoring. The positive predictive values for the Apple and Huawei technologies were 0.84 and 0.916, respectively.

There are numerous mobile software applications that utilize smart watch or smart phone flash and camera sensor to assess pulse rate variability, but accuracy is usually tested in restricted conditions. Clearly, diagnosing AF requires very high specificity to avoid situations where patients are treated with lifelong anticoagulative medications and suffer bleeding risk as a result of incorrectly diagnosed AF. The measured PPG should only be used as an indication for further evaluation: ECG visually assessed by a qualified doctor is required for initial diagnosis. However, PPG monitoring could be efficacious in monitoring the AF burden on patients with already diagnosed AF or after catheter ablation (6).

### 4.2. Electrocardiogram

The results of the ECG performance of the wrist device (sensitivity 97.7% and specificity 89.8%) were also on par with the results in the recent literature. Hermans et al. compared long-term intermittent AliveCor Kardia recording including automatic analysis to Holter heart rhythm monitoring for the detection of AF recurrence after cardiac ablation therapy in 115 patients. The patients made 30-s ECG recordings three times a day and whenever experiencing symptoms during a 4-week period. The sensitivity obtained was 95.3% and the specificity 97.5% (36). Karregat et al. invited 205 primary care patients aged ≥65 years with a negative 12-lead ECG to wear a Holter monitor for 2 weeks and to use a MyDiagnostick single-lead ECG device three times a day for 60 s ECG recordings. The sensitivity and specificity results of AF detection were 66.7 and 68.8%, respectively, (37). Svennberg et al. used the Zenicor device in an AF screening study on 3,209 persons. The study did not have a reference device, but the performance of the ECG analysis algorithm used by Zenicor was compared to manual interpretation of the same ECGs. The outcomes were 97.8% sensitivity and 88.2% specificity (38). The AliveCor Apple iPhone cover with Lead I ECG measurement (iECG) was used in two long-term studies by two different groups. The number of patients in the studies were 60 and 42 and the study durations were 1 and 4 weeks. In the first study, the reference methods were a cardiologist’s interpretation of the iECG in combination with the noise-reduced iECG, and 12-lead ECG or Holter monitoring. The second study used the Pacetrack transtelephonic monitor to record ECG. The respective sensitivity and specificity results were 100 and 97% (39), and 94.6 and 92.9% (40).

Ten recent short-term inpatient studies were also reviewed. As with the PPG short-term studies, these provide a snapshot of the patient’s situation and provide limited information on the performance in the actual use environment. Median of the ECG recording duration was 30 s, median number of patients 144 and median sensitivity 93.0% (range 75.0–100.0%) and specificity 95.0% (84.0–95.7%) (41–50).

In the present study, the wrist device ECG was recorded using stainless steel dry electrodes. Ten percent of the ECG segments were labeled as inadequate quality by the algorithm with an additional 5 and 1% by the cardiologists. The physicians’ subjective judgment on the adequacy of the quality of the ECG records for rhythm assessment was based on their prior experience with single lead ECG interpretation and the clinical context. In the Holter using disposable Ag/AgCl electrodes, 10% of the data were assessed as low quality due to artifacts. The stainless steel dry electrodes can record diagnostic level data, and in long-term use they compare with the Holter data quality. However, it must be noted that due to the ECG recording method of the wrist device, subjects are instructed to remain still during measurement, whereas the continuous Holter data includes both low activity and high activity periods.

There is usually a trade-off between sensitivity and specificity when monitoring a medical condition. Technical adjustments in algorithms or methods toward high sensitivity may cause lowered specificity and vice versa as can be seen in our study when comparing the performance in the cases where (A) only the label *Possible Arrhythmia* or (B) any cardiac event labeled by the algorithm was accepted as positive prediction (Tables 3, 4). However, diagnosis never relies solely on just algorithm classification. A trained medical professional always reviews the data before treatment decisions are made.

In a device with its intended purpose to detect AF it is important to optimize for high sensitivity to correctly detect all patients with AF. False positives may lead to further unnecessary investigations but constitute a lower burden on society than false negatives, which can cause strokes with high treatment and rehabilitation costs and in the worst-case lead to patient death. It must be noted, however, that only AF can be reliably diagnosed using Lead I ECG. Depending on the subject the flutter waves of AFL cannot necessarily be distinguished in Lead I. This is because during AFL Lead I is in most subjects low amplitude or isoelectric for the atrial activity (51). Further, more ECG channels are needed to diagnose other arrhythmias, but ambulatory single-lead monitoring can provide information to trigger subsequent evaluation, e.g., 12-lead ECG or Holter.

The coronavirus pandemic that started in late 2019 has restricted travel and accelerated the use of telehealth. Wearable technology affords an opportunity for continuous heart rhythm assessment. The increasing popularity of wearable technology capable of detecting AF alongside the development of direct acting oral anticoagulants has also sparked new research interest. Could patients with paroxysmal AF under continuous heart rhythm monitoring be treated with direct acting oral anticoagulants and exposed the risk of bleeding risk only intermittently when a sufficiently long period of AF is detected with by wearable device (52)? Obviously, this “pill-in-the-pocket” anticoagulation strategy still requires rigorous clinical investigation.

However, the use of smart devices to monitor heart rhythm may cause inequality among patients as these devices are not usually integrated into national health care systems or reimbursed, which makes them more readily available to people with better economic status and the high cost may impede their use.

The usual problem with consumer smart devices capable of cardiac rhythm monitoring is that their use is focused on the young and on those with high socioeconomic status and advanced interest in their health already, but the risk of AF starts to rise after the age of 55 (5). Smart devices have varied ECG recording methods. In those worn on the upper limb the contralateral limb is brought into contact with the device to record Lead I. In many devices, a crown button at the side of the device is pressed with a finger to form an electrical circuit for the recording. In the wrist device investigated, the recording is done by covering the whole anterior surface of the device where the dry electrodes are located with the palm of the opposite hand. This recording method may be easier for elderly users and the large skin-electrode contact area can even provide improved ECG signal quality for some subjects. The recorded data is transferred to the cloud for interpretation either post-hoc through a computer or automatically with a separate data gateway device. The gateway device can be positioned in the user’s home, and will automatically send new recordings to the cloud when the user is near the gateway. This approach may be advantageous if the healthcare delivery process is arranged so that there is someone to observe the transferred data.

The key limitation of this study was that only subjects with a prior diagnosis of AF/AFL were included as an adequate number of relevant events were needed to validate the technical performance of the proposed system. The sample size was limited so the findings cannot be generalized without caution. Depending on the study design, PPG technology may have limitations; the irregular pulse notification of Apple Watch had only 41% sensitivity for AF in subjects who had recently undergone cardiac surgery (53). The group recorded 50 patients over 2 days. On telemetry AF was observed in 90 instances, and sinus rhythm was seen in 202 instances. Twenty-five of the 50 patients had ≥1 episodes of AF. In an earlier study, Tison et al. used Apple Watch to record 51 cardioversion patients for 20 min to achieve a sensitivity of 98.0% (21). Wasserlauf et al., recorded 24 patients with a history of paroxysmal AF over a mean duration of 110 days. Eighty-two episodes of AF ≥ 1 h were detected on the implantable loop recorder while the smartwatch was being worn, and the sensitivity was 97.7% (12). The three studies had different patient populations which may account for the variation in the results. However, the seemingly poor algorithm sensitivity in (53) may result from a lack of data due to subjects who have a low AF burden combined with some difficult to detect AF episodes leading to false negatives.

The standard ECG recording obtained with wrist devices is equivalent to ECG Lead I which is sub-optimal for the detection of P-waves and flutter waves. Atypical recording configurations could provide additional lead tracings more suitable for certain arrhythmias (6, 54). However, from the user point of view taking Lead I ECG between the arms is easy and thus practical.

The system investigated could be suitable for AF screening in older age groups due to its good usability, long battery life, signal quality, and no need for a paired smart phone. Our next target is to investigate the performance of the PulseOn Arrhythmia Monitor System in a screening setting. In that study we will recruit subjects with AF risk factors but no prior diagnosis of AF or AFL. This study will also evaluate the suitability of the system for long-term use.

## 5. Conclusion

The PulseOn Arrhythmia Monitor System comprising the wrist device for PPG/ ECG recording and the Data Management Service has been validated. It was found that the system can reliably detect and diagnose AF in an ambulatory setting. The wrist device and the Data Management System were found easily usable by the study subjects and by the participating cardiologists.

## Data availability statement

The datasets presented in this article are not readily available because study sponsor PulseOn Oy holds the rights to the data that support the findings of this study and therefore the availability is restricted. The data was used under license for the current study. However, the data is available from the authors representing the sponsor upon reasonable request. Requests to access the datasets should be directed to antti.vehkaoja@pulseon.fi.

## Ethics statement

The studies involving human participants were reviewed and approved by Research Ethics Board of Tampere University Hospital. The patients/participants provided their written informed consent to participate in this study.

## Author contributions

HS: methodology, investigation, data curation, writing—original draft, and writing—review and editing. AJ: methodology, formal analysis, writing—original draft, writing—review and editing, visualization, and funding acquisition. KK: investigation, data curation, and writing—review and editing. TH: software, formal analysis, data curation, and writing—review and editing. MN: software, formal analysis, and data curation. JH: methodology, project administration, and writing—review and editing. AV: conceptualization, methodology, resources, supervision, project administration, and writing—review and editing. All authors contributed to the article and approved the submitted version.

## Funding

This study was financially supported by PulseOn Oy and partly supported by the Tampere University Faculty of Medicine and Health Technology doctoral program and the Tampere University Hospital Support Foundation, Tampere University Hospital project number MK355.

## Conflict of interest

AV, KK, TH, and MN are employees of PulseOn Oy. KK is also a minority shareholder in PulseOn Oy.

The remaining authors declare that the research was conducted in the absence of any commercial or financial relationships that could be construed as a potential conflict of interest.

## Publisher’s note

All claims expressed in this article are solely those of the authors and do not necessarily represent those of their affiliated organizations, or those of the publisher, the editors and the reviewers. Any product that may be evaluated in this article, or claim that may be made by its manufacturer, is not guaranteed or endorsed by the publisher.
